# Prenatal diagnosis of a 46,XY karyotype female fetus with an SRY-associated gonadal dysgenesis, conceived through an intracytoplasmic sperm injection: a case report

**DOI:** 10.1186/s12884-022-04431-6

**Published:** 2022-02-05

**Authors:** Lidiia Zhytnik, Maire Peters, Kadi Tilk, Tiia Reimand, Piret Ilisson, Tiina Kahre, Ülle Murumets, Aivar Ehrenberg, Eva-Liina Ustav, Neeme Tõnisson, Signe Mölder, Hindrek Teder, Kaarel Krjutškov, Andres Salumets

**Affiliations:** 1 Competence Centre on Health Technologies, Teaduspargi 13, 50411 Tartu, Estonia; 2 Department of Obstetrics and Gynaecology, Institute of Clinical Medicine, University of Tartu, Tartu, Estonia; 3 Department of Clinical Genetics, United Laboratories, Tartu University Hospital, Tartu, Estonia; 4 Department of Clinical Genetics, Institute of Clinical Medicine, University of Tartu, Tartu, Estonia; 5 Women’s Clinic, Tartu University Hospital, Tartu, Estonia; 6 Institute of Genomics, University of Tartu, Tartu, Estonia; 7 Department of Reproductive Medicine, West Tallinn Central Hospital, Tallinn, Estonia; 8 Institute of Bio- and Translational Medicine, University of Tartu, Tartu, Estonia; 9 Division of Obstetrics and Gynaecology, Department of Clinical Science, Intervention and Technology (CLINTEC), Karolinska Institutet, Stockholm, Sweden

**Keywords:** ICSI, oligozoospermia, *SRY*, sexual development disorders, prenatal diagnosis, NIPT

## Abstract

**Background:**

Permanent progression of paternal age and development of reproductive medicine lead to increase in number of children conceived with assisted reproductive techniques (ART). Although it is uncertain if ARTs have direct influence on offspring health, advanced paternal age, associated comorbidities and reduced fertility possess significant risks of genetic disorders to the offspring.

With a broad implementation of a non-invasive prenatal testing (NIPT), more cases of genetic disorders, including sex discordance are revealed. Among biological causes of sex discordance are disorders of sexual development, majority of which are associated with the *SRY* gene.

**Case presentation:**

We report a case of a non-invasive prenatal testing and ultrasound sex discordance in a 46,XY karyotype female fetus with an *SRY* pathogenic variant, who was conceived through an intracytoplasmic sperm injection (ICSI) due to severe oligozoospermia of the father.

Advanced mean age of ICSI patients is associated with risk of *de novo* mutations and monogenic disorders in the offspring. Additionally, ICSI patients have higher risk to harbour infertility-predisposing mutations, including mutations in the *SRY* gene. These familial and *de novo* genetic factors predispose ICSI-conceived children to congenital malformations and might negatively affect reproductive health of ICSI-patients’ offspring.

**Conclusions:**

Oligozoospermic patients planning assisted reproduction are warranted to undergo genetic counselling and testing for possible inherited and mosaic mutations, and risk factors for *de novo* mutations.

## Background

With routine availability of non-invasive prenatal testing (NIPT), the rate of prenatally diagnosed disorders of sex development (DSDs) has multiplied owing to sex discordance between fetal chromosome and ultrasound analyses. DSDs are a heterogenous group of rare congenital conditions, characterized by genetic (e.g. sex chromosomes’ abnormalities, single nucleotide variants in autosomal and sex-linked genes), gonadal and anatomic sex aberrations with an estimated prevalence of 1 in 2000 live births. An early diagnosis of DSDs has ample importance for a patient’s lifelong care: family counselling and education prior to delivery, shared decision-making in pre- and post-puberty, and involvement of a multidisciplinary medical team to provide the best comprehensive care. Abnormalities of the sex-determining region Y (*SRY*) gene appear to be one of the predominant genetic causes of DSDs [1]. Around 15% of 46,XY females and 80% of 46,XX males harbor pathogenic variants or translocations of *SRY*, respectively.

We describe a case of a pregnancy achieved through an intracytoplasmic sperm injection (ICSI) with an ultrasound and NIPT sex discordance caused by an SRY-associated gonadal dysgenesis in a fetus. The case raises number of questions around ICSI, oligozoospermia and risk of genetic disorders, namely: (i) does oligozoospermia indicate the presence of a sperm mosaicism or a risk of *de novo* mutations (DNMs) in the offspring; (ii) do ICSI-fathers demand additional attention owing to advanced paternal age (APA) and inherent higher risk of DNM*s*; and (iii) do ICSI-pregnancies/children require an in-depth genetic analysis?

## Case presentation

A non-consanguineous Caucasian couple with an advanced parental age (40 years female and 46 years male) presenting with infertility, referred to the Women’s Clinic of the Tartu University Hospital.

**Fig. 1:**
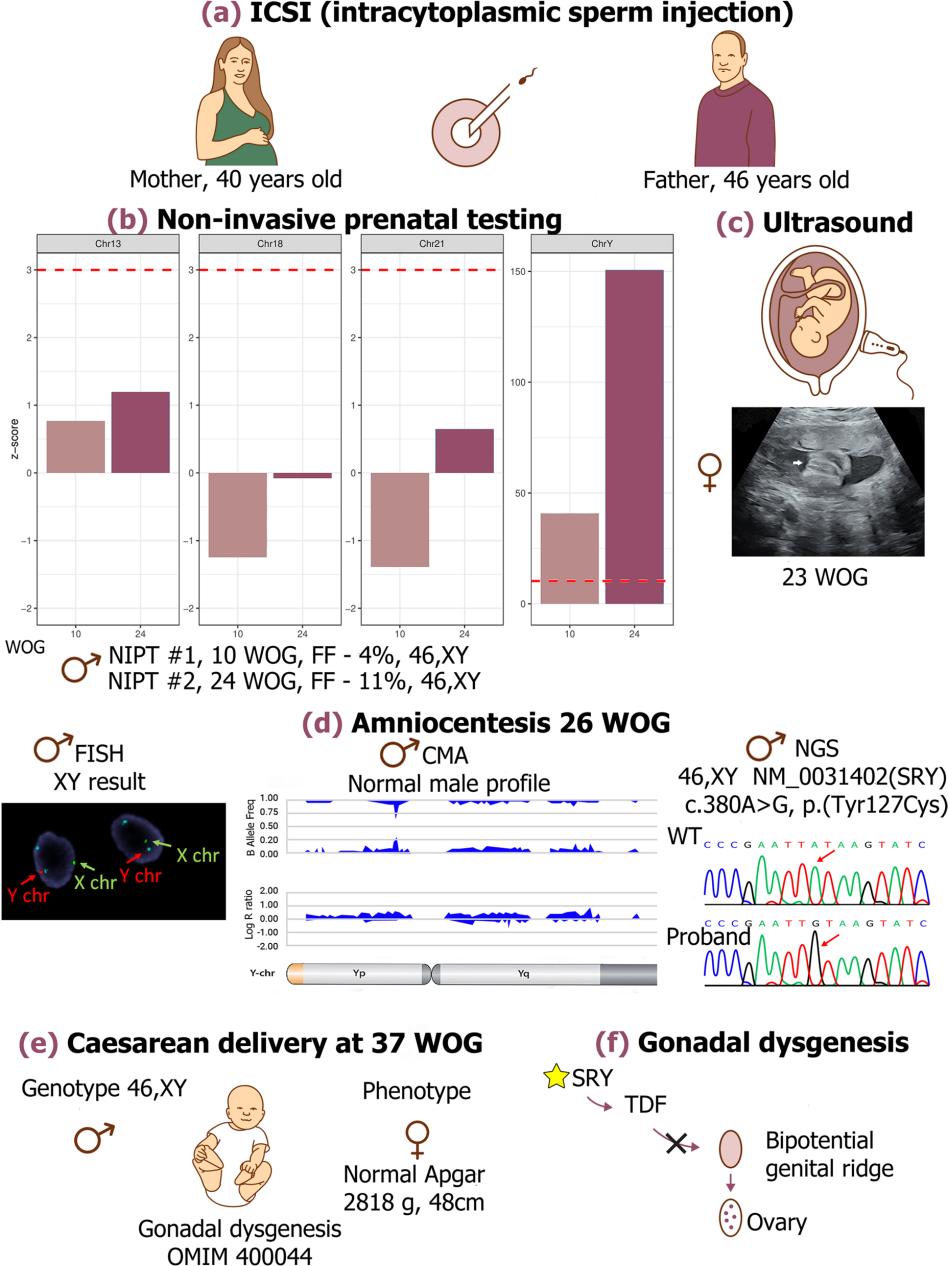
Prenatal diagnosis of a 46,XY karyotype female fetus with an SRY-associated gonadal dysgenesis, conceived through an intracytoplasmic sperm injection (ICSI). **a** A couple with advanced age referred for ICSI due to oligozoospermia. **b** The results of Non-invasive prenatal testing (NIPT) analysis revealed low risk for trisomies 13, 18 and 21 at both 10^th^ (light pink bars) and 24^th^ (dark pink bars) week of gestation (WOG). The limit of low-risk score is indicated by a red dotted line. In case of Y-chromosome, the red dotted line indicates the z-score from which the fetus is designated as male. **c** Ultrasound investigation showed a female fetus at 23 weeks of gestation. **d** Interphase nuclei from uncultured amniocytes by Fluorescence in situ hybridization (FISH) shows one green (X chromosome), one red (Y chromosome) and two blue (18 chromosome) signals. Chromosomal microarray (CMA) revealed normal male profile. Sequencing (NGS) revealed a hemizygous variant c.380A>G(p.(Tyr127Cys)) on the *SRY* gene. **e** A child with a female phenotype was born on 37 weeks of gestation. A child was diagnosed with gonadal dysgenesis (OMIM 400044). **f** In current case, the pathogenic variant in *SRY* caused altered functioning of a testis determining factor (TDF) and bipotential genital ridge developed as ovaries, resulting in female phenotype in a 46,XY child. The Fig. 1 is an origin image created specifically for the current article using Adobe Photoshop CC 2020

The female has had one spontaneous pregnancy with previous partner, which resulted in a birth of a healthy child. The male did not have children from previous relationships. In the current relationship the couple has been suffering from infertility. Analysis of the semen revealed a severe oligozoospermia (3 × 10^6^ spermatozoa/ml). As the medical history of the man was negative for any chronic infertility associated conditions, the reason for oligozoospermia remained unexplained, and the couple was recommended ICSI. In 2016 a phenotypically healthy daughter was born from an ICSI-conceived pregnancy. In 2020, the couple was again recommended ICSI (Fig. 1a). All three retrieved oocytes were successfully fertilized, two embryos were transferred, resulting in a singleton pregnancy.

NIPT for the second pregnancy was performed at the 10 weeks of gestation (WOG) due to an increased maternal age-associated risk of trisomy 21. Venous blood was collected from the mother into a cell-free DNA blood collection tube (Streck, USA). The sample was sent to the Competence Centre of Health Technologies (Estonia), processed and sequenced according to a previously described protocol [2]. The aneuploidy status, fetal fraction (FF), and fetal sex were analyzed using NIPTmer software and NIPTIFY test [3, 4]. The results indicated a low risk for trisomies 13, 18 and 21, and a male fetal sex (FF 4%) (Fig. 1b).

Both at 20 and 23 WOG, fetal ultrasound investigations showed a female fetus (Fig. 1с). Due to sex discordance, the NIPT test was repeated at the 24th WOG (FF 11%). The result of the second NIPT was in accordance with the first NIPT analysis, indicating the presence of a Y chromosome. A signal of the Y chromosome was additionally assessed by the normalized difference between the detected coverage and the expected coverage of the Y chromosome in case of a female fetus, which increased 3.6 times, indicating correlation with the FF increasing from 4 to 11% (Fig. 1b).

Owing to sex discordance in NIPT and ultrasound results, amniocentesis was performed at the 26th WOG. The fluorescence in situ hybridization (FISH) analysis on amniotic fluid cells using DNA probes (OGT/Cytocell, UK) specific for chromosomes 13, 18, 21, X and Y showed a normal non-mosaic male pattern without aneuploidies (Fig. 1d). Chromosomal microarray (CMA) from fetal DNA revealed male, 46,XY, profile with one single copy gain on the interstitial region of chromosome 16 short arm. A 2.66 Mb in size duplication of chromosomal region from 16p13.11 to 16p12.3 was present (GRCh37 16:15,493,046-18,156,351). The duplication of a 16p13.11 recurrent breakpoint region (BP2-BP3) has unknown clinical significance, according to the ClinGen Dosage Sensitivity Curation Page.

Sequencing with Illumina TruSight One Expanded gene panel (6,700 genes, on average 20× coverage for 94.1% of targeted regions) from amniotic cells revealed a hemizygous variant NM_003140.2(SRY):c.380A>G(p.(Tyr127Cys)) on *SRY* (Fig. 1d). The average coverage of the *SRY* gene was 51×, and for the NM_003140.2(SRY):c.380 position 57x. The c.380A>G *SRY* variant is reported as pathogenic by the ClinVar and The Human Gene Mutation Database (HGMD) Professional, and associated with gonadal dysgenesis (OMIM 400044). The variant is absent from gnomAD database and is present in the dbSNP (rs104894973). No data is available on the frequency of the variant in the population. Duplication of a 16p13.11-16p12.3 chromosomal region was seen. Owing to the unavailability of parental samples, a *de novo* nature of variants found in the proband was not confirmed. However, follow up analysis revealed the presence of the same 16p13.11-16p12.3 duplication in the proband’s older sister , who had a normal female profile, indicating that this microduplication was likely inherited from one of the parents.

A child with a female phenotype was born at 37 WOG by Caesarean section because of a fetal distress. No abnormalities were noticed in the otherwise healthy newborn, weight 2,818g, height 48cm, and Apgar score indicating that the newborn was in good health (Fig. 1e).

## Discussion and conclusions

Despite uncertain genetic risks to offspring, the number of ICSI procedures (sometimes unjustified) constantly grows worldwide. By overcoming biological barriers, ICSI increases the risks of congenital malformations and continuously reduces offspring’s reproductive health. As sex chromosome numerical abnormalities and autosomal structural rearrangements cause infertility, it was proposed that in males presenting with sperm concentration less than 10 x 10^6^ spermatozoa/ml karyotype analysis should be performed prior to ICSI [5]. However, to avoid transmission of genetic anomalies to ICSI-children, germline point mutations should also be comprehensively assessed. Firstly, ICSI patients might harbour mutations predisposing to infertility, including also in *SRY* gene [6]. Secondly, as mean age of ICSI-fathers is >40 years, there is an APA-associated risk of DNMs and monogenic disorders (MDs) in the offspring [7–9]. The presented case confirms necessity of in-depth screening along with basic karyotyping analysis in parents referred to ART procedures. Analysis of chromosomal abnormalities and single nucleotide variants associated with advanced age of parents has potential to reduce the burden of genetic conditions in the offspring conceived through ART.

*SRY* codes for a testis determining factor (TDF), which is a key transcription factor in male sex differentiation. In the presence of *SRY*, a genital ridge develops as testes; however, if *SRY* expression is absent or the mutated protein is non-functional, a bipotential genital ridge will develop as ovaries (Fig. 1f). *SRY* pathogenic variants affect DNA binding, bending or cellular localization of the TDF. Impaired nucleus transportation of the TDF and failure of a transcriptional complex assembly result in a disrupted sex differentiation cascade [10]. Patients with *SRY*-associated complete gonadal dysgenesis commonly have a female phenotype. However, they do not develop secondary sexual characteristics and have primary amenorrhea. As a rule, the disorder is non-syndromic and no abnormalities in other organ systems are present at birth. The phenotype spectrum of *SRY*-associated gonadal dysgenesis is extremely variable, presenting individuals with both sex phenotypes, including fertile males. A case of a familial *SRY* pathogenic variant (c.380A>T, p.(Tyr127Phe)) with different substitution of the same nucleotide as reported in our patient (c.380A>G(p.(Tyr127Cys)), shared by normal fertile 46,XY father and an affected 46,XY daughter with female phenotype has been reported [11, 12]. Moreover, cases of paternal germline mosaicism for *SRY* variants were described, resulting in an *SRY*-associated gonadal dysgenesis shared by siblings [13]. However, as the older sister had a normal female profile, and taking into consideration APA (46 years) and severe oligozoospermia of the patient’s father, additional studies are needed to rule out the possibility of a familial variant or paternal germline mosaicism. Unfortunately, parents refused from further analysis. Nevertheless, a possibility of a *de novo* variant cannot be ruled out, as ICSI overcomes biological barriers, increasing risks of *de novo* and familial genetic anomalies and reducing reproductive health, compared to children conceived spontaneously [14, 15].

DSDs, including gonadal dysgenesis, has a significant impact on patient’s life quality, increasing risks of bone fragility, ovarian cancer, gonadal neoplasia, endocrinological complications, psychological burden and causing female sterility [16]. Early diagnosis of the disorder has vast importance not only for disease management, reducing health risks, but also supports optimal psychological development of an individual with DSD, and helps to avoid irreversible medical, surgical and social decisions prior to fully informed decision making. Due to health risks affecting genitourinary and endocrine systems, which accompany DSD, early disease management by a multidisciplinary team is highly important. Congruence of a gender identity with a sex of rearing is especially important for the emotional wellbeing and quality of life of individuals with DSD [17]. Due to the presence of a female external genitalia, individuals with complete gonadal dysgenesis are usually reared as females and develop mainly female gender identity. Even a successful pregnancy can be achieved by patients using donor oocytes. However, with a broader use of NIPT, many cases of DSD are being discovered prenatally, shaping a new agenda for psychosexual studies of DSD individuals and parental reactions on DSD and choices of sex rearing.

As a non-invasive procedure, NIPT has become a favorable way of prenatal screening, including evaluation of sex chromosomes. Fetal sex detection specificity of NIPT is 99.6%, and for ultrasound analysis a phenotypical sex evaluation rates between 98-100% [1]. The case shows the importance of revealing of NIPT and ultrasonography fetal sex determination results to all involved parties (e.g., parents, clinicians, NIPT specialists). Although 34% of sex discordance cases may be caused by human error, like samples’ mislabelling or limitations of an ultrasound during early WOG, biological causes of sex discordance should not be underestimated [18]. Sex discordance might arise due to various pathological states, and thus need to be carefully assessed. Previously reported cases revealed a whole spectrum of DSDs involved in NIPT and ultrasound sex discordance. 9p deletion syndrome (XY genotype, female phenotype) with deletion of *DMRT1* and *DMRT3* genes aligning in the deleted region resulted in a male fetus sex reversal. Similarly, male fetus sex reversal was associated with a case of complete androgen insensitivity syndrome (XY genotype, female phenotype) with pathogenic variants in the *AR* gene inherited from asymptomatic 46,XX mother. Yp:Xp translocations (XX genotype, male phenotype) resulted in the short arm of the Y chromosome (including *SRY*) translocated onto the short arm of the X chromosome and presence of a male phenotype in a fetus with female genetic profile. Other cases represented mosaic monosomy X/XY and Yp11.2 gains [19]. Keeping in mind that *SRY* anomalies are one of the primary genetic causes of sex reversal, inclusion of *SRY*, as well as the most common DSD genes (i.e., *NR5A1, MAP3K1*) into NIPT panels for MDs, might benefit families declining amniocentesis. For now, DSD genes are not covered by the most popular NIPT panels like GeneSafe^TM^ (UK) and Natera Vistara (USA).

The case stresses the importance of sex determination via NIPT and its comparison with results of ultrasonography sex identification. Relatively high prevalence of DSDs also supports the inclusion of the most common DSD genes into extended NIPT panel for MDs.

Importantly, to decrease risks of congenital malformations in the offspring, greater attention should be paid to genetic risk factors in males with severe oligozoospermia prior to ICSI, along with in-depth genetic analysis of ICSI-pregnancies/children.

## Data Availability

The datasets used and/or analysed during the current study are available from the corresponding author on reasonable request.

## Abbreviations

APA: Advanced Paternal Age
ART: Assisted Reproductive Techniques
CMA: Chromosomal Microarray Analysis
DNMs: *De Novo* Mutations
DSDs: Disorders of Sex Development
FF: Fetal Fraction
FISH: Fluorescence *In Situ* Hybridization
HGMD: The Human Gene Mutation Database
ICSI: Intracytoplasmic Sperm Injection
MDs: Monogenic Disorders
NIPT: Non-Invasive Prenatal Testing
*SRY*: Sex-determining Region Y
TDF: Testis Determining Factor
WOG: Weeks of Gestation
